# Intestinal Myiasis Caused by *Sarcophaga* spp. in Cusco, Peru: A Case Report and Review of the Literature

**DOI:** 10.1155/2018/3685439

**Published:** 2018-05-27

**Authors:** Priscilla Ly, Adiel Aizenberg, Taylor Martin, Martha Lopez, Miguel Arturo Saldaña, Grant Leslie Hughes, Miguel Mauricio Cabada

**Affiliations:** ^1^School of Medicine, University of Texas Medical Branch, 301 University Boulevard, Galveston, TX 77555, USA; ^2^Cusco Branch, Tropical Medicine Institute, Universidad Peruana Cayetano Heredia, Calle Jose Carlos Mariategui J-6, Wanchaq, Cusco, Peru; ^3^Department of Microbiology and Immunology, University of Texas Medical Branch, 301 University Boulevard, Galveston, TX 77555, USA; ^4^Department of Pathology, University of Texas Medical Branch, 301 University Boulevard, Galveston, TX 77555, USA; ^5^Division of Infectious Diseases, Department of Internal Medicine, University of Texas Medical Branch, 301 University Boulevard RT 0435, Galveston, TX 77555, USA

## Abstract

Myiasis is the infestation by dipterous fly larvae in humans and animals. The larvae can infect living or necrotic tissue involving the skin, nasopharynx, genitourinary, and gastrointestinal tracts. The accidental ingestion of eggs causes infection of the intestinal tract. We report a case of intestinal myiasis caused by *Sarcophaga* spp. larvae in a two-year-old child from Limatambo province in the Cusco region of Peru. Live larvae were identified incidentally in this child's stool sample during the study screening for *Strongyloides stercoralis*. The child did not have any constitutional or abdominal symptoms. The morphological examination of the specimen under magnification revealed *Sarcophaga* spp. larvae. We performed a literature review of publications reporting intestinal myiasis caused by *Sarcophaga* spp. and discussed key aspects of this infestation.

## 1. Introduction

Myiasis is the infestation with dipterous fly larvae of humans and other mammals, typically livestock [1]. Myiasis is a natural infection of livestock causing a significant burden on this industry. Occasionally, humans exposed to endemic areas suffer from zoonotic fly larvae infestations [2]. The fly larvae feed on living or necrotic host tissue, bodily fluids, or ingested food [3]. Human myiasis affects the skin most often and also can affect other organs, such as the nasopharynx, digestive tract, ear canal, orbits, and genitourinary tract [4]. Several classifications of myiasis have been proposed mostly depending on the anatomical location of the infestation, the stage of the larval development, or the existence of an obliged parasitic stage [1]. Most of the fly species that cause intestinal infestation are considered facultative or accidental myiasis [5]. Fly larvae that can cause a parasitic infestation but can also complete their cycle in the environment are considered facultative. Although these commonly cause wound or necrotic tissue myiasis, eggs or larvae in the environment can be accidentally ingested causing intestinal myiasis [6]. The diagnosis is confirmed when the offending larvae are passed in one or more consecutive stool samples [7]. Intestinal myiasis is generally transient and asymptomatic, though symptoms may include nausea, vomiting, and abdominal pain [8, 9]. Intestinal myiasis caused by the larvae of the flesh fly *Sarcophaga* spp. is reported in several countries, but to date no case has been reported in Peru. We report a case of intestinal myiasis caused by *Sarcophaga* spp. in the highlands of Peru and present a review of the literature about intestinal myiasis.

## 2. Case Presentation

A two-year-old male from Sauceda, a rural community of Limatambo district (elevation 2,550 meters) in the Cusco region of Peru, presented with four white, mobile larvae in his stool measuring approximately 12 mm. This child was a participant in a community study evaluating the prevalence of *Strongyloides stercoralis* infection. Participants in this study were instructed to collect freshly produced stool samples directly in clean plastic containers and to close them with a hermetic lid. The stool samples were immediately tested by the agar plate culture method. This method uses a Petri dish with a nutritive agar in which a portion of the stool sample is placed. Then a lid is taped hermetically to prevent *Strongyloides* larvae from escaping, and the dish is incubated to observe the track left by *Strongyloides* larvae in the agar. The fly larvae were discovered incidentally in the agar culture plate of this child. The patient was previously healthy, and the mother denied any symptoms including fever, nausea, vomiting, diarrhea, and abdominal pain. He did not have any apparent skin wounds on his body. The mother reported that the child's diet consisted of poultry, fruit, and vegetables. He lived with several extended family members in an adobe house with dirt floors and opened windows. Several animals including dogs, cats, ducks, and chickens roamed freely in and out of the household. The house was largely infested with flies.

**Figure 1:**
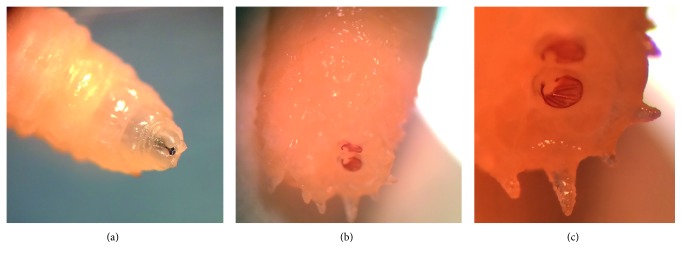
(a) Anterior end of the *Sarcophaga* spp: larva with two oral hooks. (b) Posterior spiracles located deep inside a fossa with surrounding tubercles. (c) Three parallel slits surrounded by an incomplete peritreme.

Morphological examination of the organism revealed features consistent with L3 larvae of *Sarcophaga* spp. The larva had smooth body segments with a broad posterior end and a tapering anterior end with two oral hooks and mouth brushes (Figure 1(a)). The posterior spiracles were located deep inside a fossa, surrounded by more than 10 tubercles (Figure 1(b)). The hidden spiracles had the characteristic findings of the genus *Sarcophaga*. The posterior spiracles consisted of three parallel slits surrounded by an incomplete peritreme, with the inner slit directed away from the median line ventrally (Figure 1(c)). These findings are supported by the diagnostic criteria of *Sarcophaga* spp. reported in the literature [10, 11].

## 3. Discussion

Several species of dipterous larvae including *Sarcophaga* spp. are capable of producing intestinal myiasis [8]. In most cases, the infection is caused by accidental ingestion of eggs laid on exposed food [12]. As contaminated food passes through the alimentary tract, eggs will hatch, and released larvae inhabit the lower gastrointestinal tract before being passed in the stool [12]. *Sarcophaga* larvae, the genus of the flesh fly family, are equipped to feed from tissue and can cause damage to the intestinal mucosa accounting for the symptoms reported in some patients [13]. The infestation is self-limited in most cases because larvae are excreted in the stool, where they are often found alive. This infestation does not allow for reproduction of larvae inside the host; however, with repeated ingestion of eggs, protracted cases have been reported with patients passing larvae for months or years [14]. Intestinal myiasis should be differentiated from pseudomyiasis, in which the patients ingest larvae rather than eggs and then pass intact larvae in the stool [15]. The larvae in pseudomyiasis are passed dead and never truly colonize the intestinal tract [15]. Larva can also be deposited in fecal samples left uncovered and typically present at earlier stages of development upon examination.

Our patient presented with multiple, living, L3 larvae in his stools and thus likely contracted intestinal myiasis from accidental ingestion of eggs. He lived in an adobe house from a rural community in close contact with several animals. Poor hygienic practices, lack of refrigeration of food, the proximity to a variety of farm animals, and open dwelling allowed for potential sources of infection in our patient [16]. In rural areas, domestic animals like dogs and cats are sometimes infested with fly larvae and can be a source for infection in children [17]. There have been reports of intestinal myiasis caused by ingestion of over ripened fruits such as pears or bananas [18, 19]. Drinking contaminated water has also been reported as a source of infection [20].

Several cases of intestinal myiasis caused by *Sarcophaga* spp. have been reported in the literature (Table 1). The four species identified include *Sarcophaga crassipalpis, Sarcophaga peregrina, Sarcophaga haemorrhoidalis,* and *Sarcophaga bullata*. The age of patients reported varies widely from 8 months to 66 years, being the average 33 years at presentation. The flesh fly belonging to the Sarcophagidae family has worldwide distribution [8]. Cases have been reported from Japan, India, Egypt, and the United States. The signs and symptoms of presentation are nonspecific and vary between reports, with some patients being asymptomatic like in this child's case [13]. *Sarcophaga* species are generally present in rural and urban environments and are commonly found in houses and indoor dwellings [25]. Our review revealed that living in rural areas and the ingestion of contaminated food products were factors that authors associated with infestation by *Sarcophaga* spp.

Intestinal myiasis can be largely benign or cause severe clinical symptoms, depending on the larval species, number, and location within the digestive tract [14, 26]. In some cases, like ours, larvae can be passed out in feces without causing many symptoms [14]. Colonic washes with polyethylene glycol have been reported to relieve gastrointestinal symptoms and eliminate intestinal larvae almost immediately [14]. Treatment with antihelminthic medications such as albendazole has not shown to improve symptoms in patients [8, 9, 14]. Education on good food-handling practices and avoiding the consumption of food products exposed to flies is important for prevention of this disease [14]. No reports of oral ivermectin use have been published in intestinal myiasis, but this might be a therapeutic option in severe cases.

In conclusion, intestinal myiasis caused by *Sarcophaga* spp. is a rare occurrence in humans that is often self-limited. Patients may have a range of presentations going from asymptomatic to nonspecific abdominal symptoms. Intestinal myiasis can pose a diagnostic challenge for physicians unfamiliar with the condition. Education on good food handling is advised to prevent reinfection.

## Figures and Tables

**Table 1:** Human cases of intestinal myiasis caused by *Sarcophaga* spp. reported in the literature.

Author/year	Species	Age	Sex	Country	Exposure	Symptoms
Shiota et al. 1990 [13]	*Sarcophaga crassipalpis*	4	Male	Japan	Contaminated food	Abdominal pain, fever, vomiting, and diarrhea

Nagakura et al. 1991 [18]	*Sarcophaga crassipalpis*	2	Female	Japan	Contaminated food	Abdominal pain
	*Sarcophaga peregrina*	38	Male	Japan	Contaminated food	Asymptomatic

Tachibana et al. 1987 [21]	*Sarcophaga peregrina*	Unknown	Unknown	Japan	Contaminated food	Asymptomatic

Hasegawa et al. 1992 [22]	*Sarcophaga peregrina*	8 months	Female	Japan	Unknown	Change in behavior and bloody stools

Udgaonkar et al. 2012 [14]	*Sarcophaga haemorrhoidalis*	25	Male	India	Unknown	Abdominal pain, diarrhea, and flatulence
	*Sarcophaga haemorrhoidalis*	35	Male	India	Unknown	Abdominal pain and flatulence
	*Sarcophaga haemorrhoidalis*	28	Male	India	Unknown	Abdominal pain and flatulence
	*Sarcophaga haemorrhoidalis*	30	Male	India	Living in a rural area	Abdominal pain, flatulence, and generalized weakness
	*Sarcophaga haemorrhoidalis*	38	Male	India	Living in a rural area	Abdominal pain and flatulence

Watson 1942 [23]	*Sarcophaga bullata*	37	Female	United States	Unknown	Flatulence, generalized weakness, loss of appetite, and weight loss
	*Sarcophaga bullata*	66	Female	United States	Unknown	Generalized weakness, loss of appetite, and weight loss
	*Sarcophaga bullata*	43	Male	United States	Unknown	Abdominal pain, flatulence, and generalized weakness
	*Sarcophaga bullata*	51	Male	United States	Unknown	Abdominal pain, flatulence, constipation, and loss of appetite

Kenney et al. 1976 [24]	*Sarcophaga* spp.	60	Male	United States	Unknown	Unknown

Ahmad et al. 2011 [25]	*Sarcophaga* spp.	10	Male	Egypt	Living in a rural area	Abdominal pain, loss of appetite, nausea, vomiting, diarrhea, and bloody stools

Das et al. 2010 [8]	*Sarcophaga* spp.	25	Male	India	Unknown	Asymptomatic

Watanabe et al. 2016 [4]	*Sarcophaga* spp.	61	Male	Japan	Contaminated food	Diarrhea

## Data Availability

Data will be freely available through the corresponding author upon request.
